# A study on the influencing factors of exercise adherence in patients with chronic heart failure: from a configuration perspective

**DOI:** 10.3389/fpsyg.2025.1536349

**Published:** 2025-06-03

**Authors:** Jing Lu, Jie Wang, Zhanhong You, Qin Wang, Guozhen Sun

**Affiliations:** ^1^School of Nursing, Nanjing Medical University, Nanjing, China; ^2^Department of Cardiology, The First Affiliated Hospital of Nanjing Medical University, Nanjing, China

**Keywords:** exercise adherence, heart failure, configuration, fsQCA, COM-B

## Abstract

**Objective:**

The study aims to investigate the configuration path of influencing factors of exercise adherence in chronic heart failure (CHF) patients based on the Capability, Opportunity, Motivation-Behavior (COM) model.

**Background:**

The effectiveness of exercise rehabilitation in patients with CHF depends upon their sustained adherence over the long term. Exercise adherence is influenced by a combination of antecedent conditions; however, the mechanisms underlying adherence to exercise in CHF patients remain unclear.

**Design:**

Cross-sectional study.

**Method:**

Convenience sampling was used to recruit patients with CHF from 3 tertiary-level hospitals in China, between January and May 2024. Latent profile analysis (LPA) was conducted to identify the latent profile of exercise adherence in patients with CHF. Fuzzy-set qualitative comparative analysis (fsQCA) was then employed by conducting necessity and sufficiency analysis.

**Results:**

A total of 219 patients with CHF participated in the study. The LPA results categorized exercise adherence among these patients into three profiles: low (28.3%), moderate (54.3%), and high (17.4%) adherence. The necessity analysis revealed that the perception of benefits was a necessary condition for achieving high exercise adherence, whereas no singular factor was deemed necessary for low exercise adherence. Furthermore, the sufficiency analysis identified four distinct configurational pathways leading to high exercise adherence and two pathways leading to low exercise adherence.

**Conclusion:**

Patients with CHF display varied exercise adherence behaviors, influenced by a range of antecedent conditions that interact synergistically, leading to diverse adherence patterns. Enhancing intrinsic motivation is essential for improving exercise adherence in this population. The exercise adherence behaviors of patients with CHF reflect distinct preferences, suggesting that healthcare providers can implement targeted interventions based on specific configurational pathways.

**Patient or public contribution:**

In the data collection phase, patients with CHF were recruited to participate in the survey.

## Introduction

Chronic Heart failure (CHF) is a clinical syndrome resulting from structural and/or functional abnormalities of the heart. It is clinically characterized by recurrent worsening of symptoms and signs and represents the advanced and often terminal stage of various cardiovascular diseases (Khan et al., 2024). Globally, CHF affects more than 64 million individuals. In China, data from the national urban employee medical insurance database indicate that approximately 12.1 million individuals living with CHF, with 3.0 million new cases diagnosed annually (Wang et al., 2021). With an ageing population, improved survival from cardiovascular diseases, and the increasing prevalence of chronic conditions such as coronary artery disease, hypertension, and diabetes, the burden of CHF is expected to rise steadily. Consequently, mitigating the social and economic impact of CHF has become an urgent public health priority in China (Savarese et al., 2023).

Exercise-based cardiac rehabilitation (ExCR) has been shown to improve the prognosis of patients with CHF by reducing morbidity, mortality, and hospital readmissions (Davies et al., 2010). The efficacy of ExCR in combination with pharmacological therapies and device-based interventions is recognized in multiple clinical guidelines, which endorse ExCR with Class *Ι*, Level A evidence (Taylor et al., 2023). Notably, the benefits of ExCR are highly dependent on sustained adherence (O'connor et al., 2009). However, studies have shown that 40 to 91% of individuals with CHF do not engage in regular exercise, and that adherence rates reported in clinical ExCR trials remain suboptimal (Pozehl et al., 2018). A randomized controlled trial of cardiac rehabilitation among patients with CHF in China found that the implementation rates of exercise rehabilitation behavior in the control group were only 28.99 and 21.59% at 8 and 12 weeks, respectively (Huang et al., 2022). Similarly, Yang et al. demonstrated that exercise adherence among CHF patients remained low across multiple dimensions of the adherence scale (Yang et al., 2024). These findings highlight that their exercise adherence is unsatisfactory both in terms of subjective psychological engagement and objective implementation of prescribed exercise regimens. Moreover, adherence to exercise was consistently lower than that observed for other CHF-related self-care behaviors (Van Der Wal et al., 2010). Despite widespread recognition of the importance of exercise as a health behavior among CHF patients, many exhibit poor adherence, thereby increasing the risk of unfavorable clinical outcomes. It is therefore essential to elucidate the factors influencing exercise adherence in CHF patients and the mechanisms through which they exert their effects.

Drawing on the HF-ACTION study (O'connor et al., 2009), Collins and Cooper investigated the determinants affecting exercise adherence among patients with CHF. The findings indicate that clinical and demographic variables are ineffective predictors of exercise adherence. Poor social support and high barriers to exercise were associated with lower exercise time (Collins et al., 2023; Cooper et al., 2015). Alonso et al. found that attitude, self-efficacy and relapse management were the effective components of exercise adherence intervention in patients with CHF (Alonso et al., 2021). Warehime et al. suggest that perceived health status may be key to the exercise adherence. Knowledge, social support, and motivation are critical influencing factors for long-term adherence to exercise (Warehime et al., 2020). Although increasing attention has been given to factors influencing exercise adherence among patients with CHF, existing studies still present several limitations. First, many studies lack guidance from a systematic theoretical framework, resulting in a narrow focus on determinants. Second, most analyses adopt a marginal approach, assuming that independent variables exert effects in isolation, thereby overlooking the inter-dependence among factors and the potential for synergistic interactions—what could be described as “chemical reactions.” Fundamentally, this reflects a neglect of the systemic and causally complex nature of individual behavior. From a holistic perspective, exercise adherence among patients with CHF results from multiple, concurrently acting antecedent conditions. These conditions may interact in various combinations, and no single pathway is solely responsible for achieving high adherence. However, the underlying configurational mechanisms associated with exercise adherence in this population remain insufficiently explored and poorly understood.

**Figure 1:**
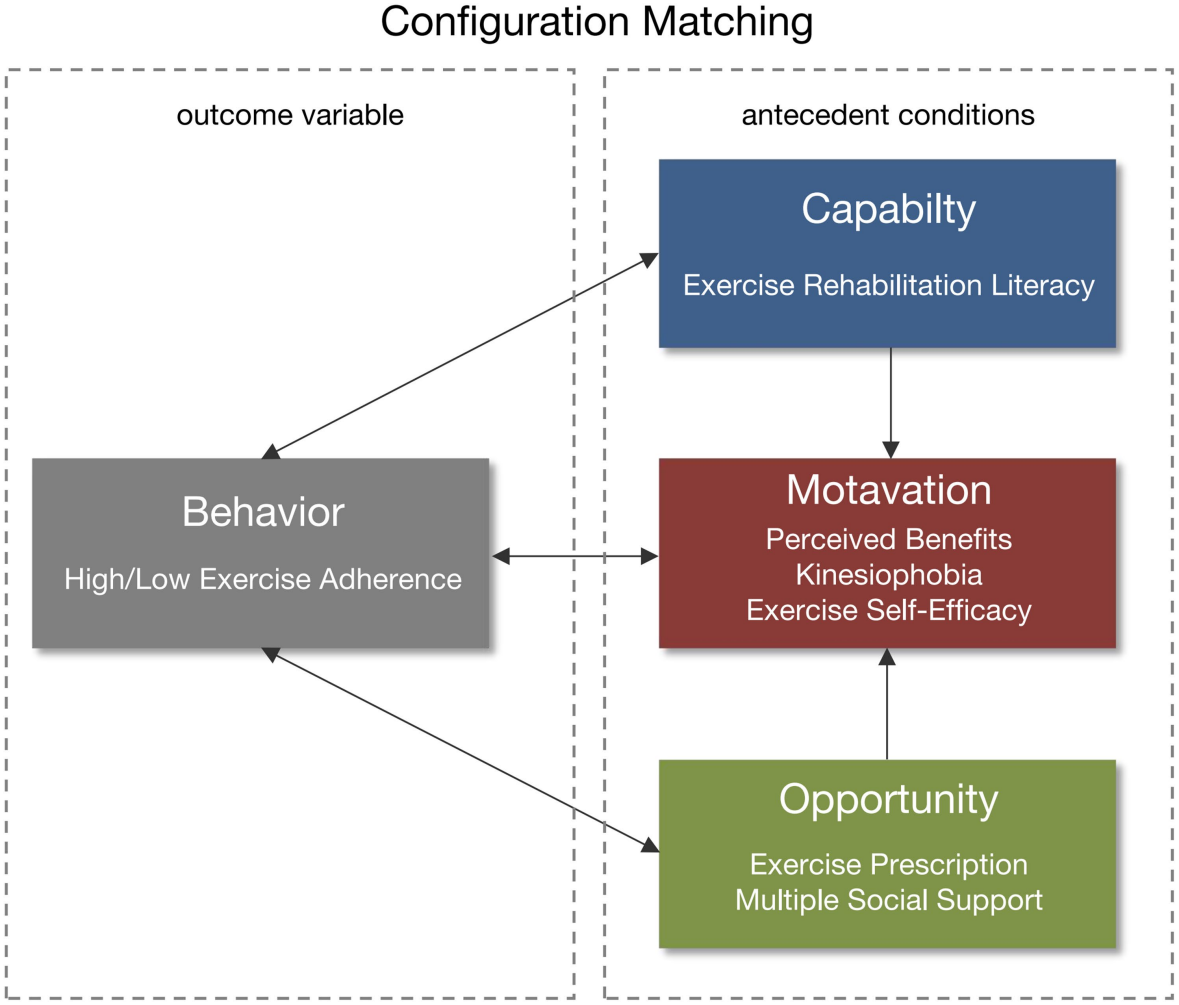
The conceptual model of COM-B.

The Capacity, Opportunity, Motivation – Behavior (COM-B) model (Michie et al., 2011), proposed by Michie et al., posits that the enactment of a behavior requires the presence of three essential conditions. Capability and opportunity can directly influence behavior or indirectly affect it through their impact on motivation. The strength of the COM-B model lies in its provision of a multidimensional analytical framework for understanding the determinants of individual behavior. In our previous research, we conceptualized exercise adherence as a behavior characterized by subjective intention (Wang et al., 2022). From the perspective of facilitating behavior change, we employed the COM-B model to comprehensively identify the influencing factors of exercise adherence in patients with CHF and constructed a corresponding measurement instrument (Gao et al., 2021; Gao et al., 2023). Specifically, the capability dimension included exercise rehabilitation literacy; the opportunity dimension comprised exercise prescription and multiple social support; and the motivation dimension encompassed perceived benefits, kinesiophobia, and exercise self-efficacy (Figure 1).

Qualitative Comparative Analysis (QCA), which was introduced by Charles Ragin in 1987, has since become a widely used method for examining causal complexity in management research (Ragin, 1987). This approach is rooted in the theoretical principles of set theory, Boolean operations, and configuration ideology. For this reason, in all QCA based methods independent and dependent variables are referred to as “conditions” and “outcomes,” respectively (Ragin, 2000, 2008, 2009). A configuration refers to a specific set of interacting conditions that, in combination, are sufficient to produce a particular outcome. QCA integrates the advantages of both qualitative and quantitative analyses, identifies conditions and outcomes from empirical data, and clarifies the underlying causal mechanisms and pathways to realization. This methodology is especially well-suited for examining the intricate interactions between conditions and outcomes they generate. The incorporation of fuzzy-set enables QCA to assess the degree of membership of each case within the set, while simultaneously maintaining the essential qualitative states of full membership and full non-membership (Ragin, 2000). In recent years, an increasing number of studies adopting fuzzy-set qualitative comparative analysis (fsQCA) have been published in the field of health. Chen et al. employed fsQCA to identify causal pathways leading to depression (Chen et al., 2024). Li et al. revealed four distinct configurations of conditions associated with physical activity behavior, demonstrating that such behavior among older adults with subjective cognitive decline results from multifactorial synergies (Li et al., 2024). Palas et al. applied fsQCA based on the extended UTAUT2 model to examine the key determinants influencing older adults’ intention and behavior regarding m-health use (Palas et al., 2022). These studies collectively illustrate the growing application of fsQCA in the health domain, particularly for uncovering complex, configurational mechanisms underlying health behaviors. Consequently, this study examined the mechanisms of exercise adherence among patients with CHF grounded in the COM-B model through the application of fsQCA. The following are the three main research questions addressed in this study: 1. Are there distinct adherence profiles of exercise adherence among patients with CHF? 2. What combination of capability, opportunity, and motivation lead to exercise adherence? 3. Does a complex and asymmetric causal relationship underlie exercise adherence in CHF patients?

## Methods

### Design and sample

This was a cross-sectional study. To estimate the sample size, a single population proportion formula ($\frac{Z_{\alpha /2}^{2}P(1-P)}{\delta^{2}}$) was used. According to the exercise adherence rate (19%) which has been reported in the HEART Camp Trial (Pozehl et al., 2018), allowable error was defined as 0.06, α = 0.05. Based on the above formula, the minimum sample size was 165. Considering the invalid questionnaire, increase the sample size by 10%, and the final sample size is 184. This study followed STROBE reporting guidelines.

### Participants

Convenience sampling was used to recruit patients with CHF from 3 tertiary-level hospitals in Nanjing, Taizhou, and Chongqing, China, between January and May 2024. The eligibility criteria were as follows, (a) aged ≥18 years; (b) met the diagnostic criteria for CHF in the 2022AHA/ACC/HFSA Heart Failure Management Guidelines (Heidenreich et al., 2022), and have been confirmed as CHF by cardiologists; (c) able to comprehend and cooperate to answer the survey questionnaires; (d) able to provide written consent and voluntary participation. The exclusion criteria were as follows, (a) Patients with absolute contraindications to exercise (Piepoli et al., 2011); (b) severe somatic diseases or limitations in exercise capacity; (c) concurrent enrollment in other clinical trials.

### Procedure

To ensure the homogeneity of the study, the protocol was further refined and finalized following investigator meetings with the principal investigator and other center staff members. Training of the data collectors was done by the principal investigator. The training content includes the purpose and significance of the study, the screening of study subjects, communication skills with subjects, the use of the questionnaire, etc. Before filling out the questionnaire, the investigators explained the study purpose, questionnaire content, and information security measures to each participant and obtained informed consent; investigators were present to explain any doubts when filling in the questionnaire; After the questionnaires were collected, investigators reviewed those questionnaires on site and immediately corrected errors.

In this study, the questionnaires with strong regularity, a large number of missing values (more than 1/3 of the total number of questions) and repeated answers were considered as invalid questionnaires and were eliminated. A total of 225 questionnaires were issued and 219 valid questionnaires were recovered, with an effective questionnaire recovery rate of 97.33%.

### Measurements

#### The general information questionnaire

The questionnaire includes items that describe participants’ sociodemographic characteristics and disease-related information, including sex, age, education level, work status, place of residence, marital status, residency, primary caregiver, monthly income, CHF duration, New York Heart Association (NYHA) class, Left Ventricular Ejection Fraction (LVEF), 6-Minute Walk Distance (6MWD).

#### Exercise rehabilitation adherence

Exercise rehabilitation adherence was measured by the Exercise Rehabilitation Adherence Scale for Patients with Chronic Heart Failure, which was developed by Gao et al. (2024). The scale consists of two dimensions: prescription adherence (five entries) and monitoring adherence (six entries). Likert 5 level scoring method was used in each entry, 1 = never, 2 = occasionally, 3 = sometimes, 4 = often, 5 = always, with the total score in the range of 11–55 points. A higher score indicates greater exercise rehabilitation adherence. The Cronbach’s *α* coefficient of the scale was 0.910.

#### Influencing factors of exercise rehabilitation adherence

Influencing factors of exercise rehabilitation adherence were measured by the scale for Measuring Influence Factors of Exercise Rehabilitation Adherence in Patients with Chronic Heart Failure, which was developed under the guidance of COM-B model by Gao et al. (2023). It is a scientific and appropriate tool for quickly and conveniently assessing the influencing factors. The scale consists of 24 entries in six dimensions: perceived benefits (three entries), kinesiophobia (two entries), exercise self-efficacy (six entries), exercise prescription (seven entries), multiple social support (three entries), and exercise rehabilitation literacy (three entries). Entries are rated on a five-point rating scale from “strongly disagree” (=1) to “strongly agree” (=5), and the kinesiophobia dimension is reverse scored. The total score is in the range of 24–120 points. The higher the score, the more positive the influencing factors of exercise rehabilitation. The Cronbach’s *α* coefficient of the scale was 0.916.

### Statistical analysis

Data were entered, cleaned, and analyzed using SPSS 26.0, Mplus 8.0, and fsQCA 3.0 software. Central tendency interpolation was used to handle missing values. Mean interpolation was used when the quantitative data were approximately normally distributed, median interpolation was used when the quantitative data were skewed, and plurality interpolation was used when the data were categorical. Continuous variables were assessed for normality using the Kolmogorov–Smirnov test. Variables that conformed to a normal distribution were presented as means and standard deviations, while those with a skewed distribution were reported as medians with interquartile ranges (IQR). Categorical variables were summarized as frequencies and percentages. Statistically significance was set at *p* < 0.05.

#### Latent profile analysis (LPA)

Latent profile analysis identifies latent subgroups of individuals within a population by focusing on individual characteristics, in contrast to “variable-centered” statistical methods that treat individuals as homogeneous or essentially homogeneous, focusing on studying combinations or developmental patterns of behavioral variables to produce more individually meaningful statistical results (Gibson, 1959). In this study, LPA of exercise adherence in patients with CHF was conducted using Mplus software. The model’s classification number was determined through a rigorous evaluation of objective adaptation indicators, resulting in a reduction of heterogeneity within each category compared to a simplistic division based on mean segmentation of individual differences.

The optimal number of profiles that fit the data was determined by comparing the fit indices of a model with k profiles to a model with k-1 profiles. Adaptation indexes including the Akaike Information Criterion (AIC), Bayesian Information Criterion (BIC), sample-adjusted Bayesian Information Criterion (aBIC), Entropy index, the Lo–Mendell–Rubin likelihood ratio test (LMR), and the Bootstrapped Likelihood Ratio Test (BLRT) (Peugh and Fan, 2013). The AIC, BIC, and aBIC fit indicators have smaller results indicating better model fit (Gabriel et al., 2015). The entropy index measures the quality of classification in LPA and takes values from 0 to 1. The closer the index is to 1, the more accurate the classification is. Clark and Muthen have shown that Entropy greater than 0.8 means correctly classified cases of more than 90% and an acceptable model (Clark and Muthén, 2009). LMR and BLRT were used to compare the fit differences between k profiles and a k-1 profile. If the corresponding *p*-value for LMR and BLRT reached significant levels (*p* < 0.05), it indicated that a model with k profiles was superior to a model with a k-1 profile (Gabriel et al., 2015).

#### Fuzzy-set qualitative comparative analysis (fsQCA)

In this study, patients’ exercise adherence was designated as the outcome variable, while the factors influencing exercise adherence were identified as antecedent conditions. Utilizing the results of the latent profile analysis, distinct configurations of adherence behaviors were classified through the application of fsQCA 3.0 software. Following the fsQCA analysis process, the data were calibrated and subjected to necessity and sufficiency analyses.

#### Ethical considerations

The study complied with the Declaration of Helsinki and was approved by the Ethics Committee of the First Affiliated Hospital of Nanjing Medical University (approval number: 2021-SR-142). All the participants signed informed consent and voluntarily participated in this study.

## Results

### Participant characteristics

In this study, 219 patients diagnosed with CHF were included. Normality testing revealed that age, LVEF, and 6MWD were not normally distributed. These variables are therefore presented as median (IQR): age was 65 (54–72) years, LVEF was 46 (34–59) %, and 6MWD was 375 (331–401) meters. The demographic and disease-related characteristics of the participants are presented in Table 1.

**Table 1:** Demographics and disease-related characteristics of the participants (*N* = 219).

Category	Items	Numbers	Percentage (%)
Gender	Male	148	67.58
	Female	71	32.42
Education level	Primary school and below	116	52.97
	Middle school	59	26.94
	High school or secondary technical school	19	8.68
	Junior college	15	6.85
	University or above	10	4.57
Work status	Working	40	18.26
	Unemployed	113	51.6
	Retirement	66	30.14
Place of residence	City	78	35.62
	Town	36	16.44
	Countryside	105	47.95
Marital status	married	189	86.3
	Unmarried	30	13.7
Residency	Living alone	32	14.61
	Living not alone	187	85.39
Primary caregiver	Himself/Herself	102	46.58
	Spouse	96	43.84
	Child	15	6.85
	Other	6	2.74
Monthly income (Chinese yuan)	<3,000	147	67.12
	3,000–5,000	47	21.46
	>5,000	25	11.42
CHF duration (year)	<1	54	24.66
	1–5	94	42.92
	>5	71	32.42
NYHA class	Ι	2	0.91
	ΙΙ	70	31.96
	ΙΙΙ	122	55.71
	ΙV	25	11.42

### Latent profile analysis

**Figure 2:**
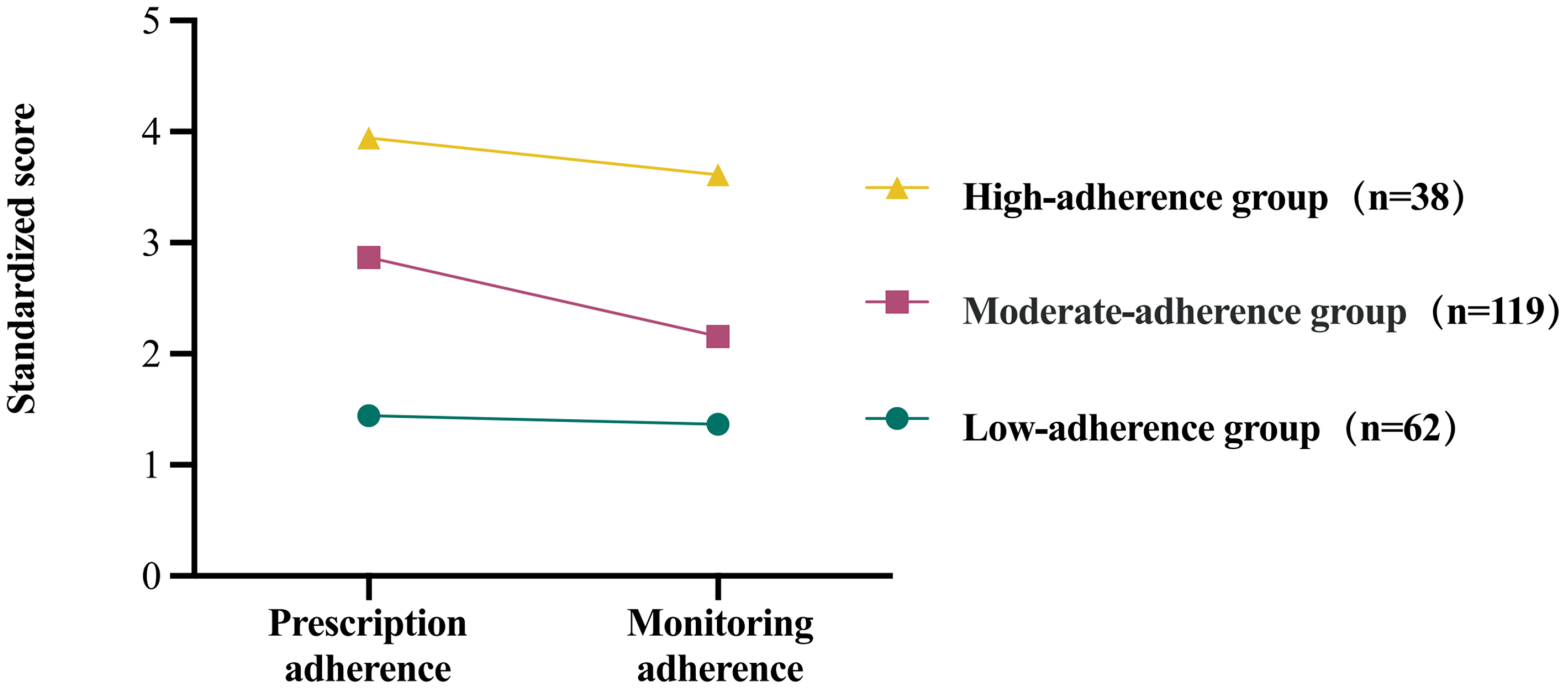
Three latent profiles of exercise adherence in CHF patients.

The two dimensions of the exercise rehabilitation adherence scale for patients with chronic heart failure, namely prescription adherence and monitoring adherence, served as the observational variables in fitting one to four latent profile models. These models were utilized to conduct a latent profile analysis of exercise adherence among CHF patients. The findings are detailed in Table 2. Compared to the two-profile model, the three-profile models demonstrated lower values for the AIC, BIC and aBIC, alongside higher entropy values. Consequently, the three-profile model was chosen for categorizing exercise adherence behaviors within this population. The probabilities of membership for each category were 28.3, 54.3, and 17.4%, respectively. In addition to model fit statistics, the average posterior probabilities for the most likely latent class membership were examined to assess classification quality. As shown in Table 3, the average posterior probabilities for the three identified profiles-low, high, and moderate exercise adherence-were 0.911, 0.930, and 0.906, respectively. These values exceed the recommended threshold of 0.70, indicating a high degree of classification certainty and supporting the reliability of the three-profile (Nagin, 2005; Stanley et al., 2016). The distinct categories were classified as low, moderate, and high exercise adherence groups based on their characteristics (Figure 2).

**Table 2:** Model fit statistics for latent profile analysis of the exercise adherence in CHF patients (*N* = 219).

Model	AIC	BIC	aBIC	Entropy	LMR (*P*)	BLRT (*P*)	Category probability
1C	1228.328	1241.884	1229.208	–	–	–	–
2C	1138.701	1162.425	1140.242	0.800	0.0000	0.0000	0.215/0.785
**3C**	**1084.666**	**1118.557**	**1086.867**	**0.802**	**0.0001**	**0.0000**	**0.283/0.543/0.174**
4C	1052.405	1096.463	1055.267	0.845	0.3745	0.0000	0.401/0.251/0.182/0.164

Bolded values indicate the optimal model selected based on comprehensive fit indices.

**Table 3:** Average latent class probabilities for most likely latent class membership.

Latent profile	C1 (%)	C2 (%)	C3 (%)
C1 (low)	0.911	0.000	0.089
C2 (high)	0.000	0.930	0.070
C3 (moderate)	0.075	0.019	0.906

### Fuzzy-set qualitative comparative analysis

The objective of this study was to examine the mechanisms underlying the manifestation of varying exercise adherence behaviors among CHF patients. Consequently, utilizing the findings from the latent profile analysis, the groups of patients exhibiting distinctly high and low levels of exercise adherence were chosen for configuration analysis.

### Calibration

In the initial step, fuzzy set calibration is applied to convert the raw data into membership score sets, which span from 0.0 to 1.0. A value of 0.0 signifies complete non-membership in a fuzzy set, while a value of 1.0 indicates full membership in a fuzzy set. Furthermore, a value of 0.5 denotes the point of greatest ambiguity in membership, indicating maximum uncertainty. In this study, the scores from the dimensions of the scales were utilized to represent the levels of the respective conditions, and the data were calibrated through the application of the direct calibration method. Based on the recommendation of Ragin (1987), the threshold for full membership threshold was identified as the 95th percentile, the crossover point was identified as the median, and the full nonmembership point was identified as the 5th percentile. The calibration values for each condition are shown in Table 4.

**Table 4:** Conditions and calibration values.

Conditions	High-adherence group			Low-adherence group		
	P5	P50	P95	P5	P50	P95
Perception of benefits	10	13.5	15	5.05	11.5	15
Kinesiophobia	2.85	5.5	8.15	2	6	10
Exercise self-efficacy	15	23.5	30	7.05	18	26.95
Exercise prescription	14	33	35	7	16.5	34
Multiple social support	8	12	15	4	9	13.95
Exercise rehabilitation literacy	9.85	12	13.15	6.1	11	14
Exercise adherence	35.85	41	48.3	11	14	20

### Necessity analysis

In accordance with the arithmetic prerequisites of fsQCA, it is imperative to evaluate the necessity of each condition before conducting the combinatorial conditional analysis. This preliminary assessment determines whether antecedent variables serve as necessary conditions for the outcome variables. Such an approach prevents the undue simplification of the subsequent truth table construction by avoiding the direct inclusion of necessary conditional variables in the truth table analysis, where they would otherwise be considered as logical remainders. Consistency serves as a criterion for assessing the necessity of the antecedent condition, while coverage indicates the extent of the antecedent condition’s explanatory power concerning the outcome. The results of the necessity analysis indicated that perception of exercise benefits constitutes a necessary condition for high exercise adherence among patients with CHF. Specifically, all patients exhibiting high adherence demonstrated a strong perception of the benefits of exercise. However, it is important to emphasize that a high perception of exercise benefits alone is not sufficient to ensure high adherence. In contrast, the consistency scores for all individual antecedent conditions related to low adherence were below the threshold of 0.90, suggesting that no single factor is sufficient to account for low exercise adherence. These findings underscore the need for further configurational analysis to identify meaningful combinations of conditions (see Table 5).

**Table 5:** Test of necessary conditions.

Conditions	High-adherence		Low-adherence	
	Consistency	Coverage	Consistency	Coverage
PB	0.934404	0.555070	0.565855	0.587401
~PB	0.501339	0.582879	0.712237	0.645122
KP	0.380857	0.734194	0.640880	0.624838
~KP	0.877510	0.433388	0.595532	0.571703
ESE	0.831325	0.702886	0.635545	0.574789
~ESE	0.732262	0.538121	0.646549	0.672330
EP	0.854752	0.519106	0.595865	0.613036
~EP	0.429049	0.478358	0.650217	0.593608
MSS	0.753681	0.626251	0.695232	0.613235
~MSS	0.719545	0.536963	0.594865	0.637143
ERL	0.766399	0.686863	0.626542	0.611256
~ERL	0.846720	0.593062	0.665222	0.638196

“~” Means logical operator NOT. PB, perception of benefits; KP, kinesiophobia; ESE, exercise self-efficacy; EP, exercise prescription; MSS, multiple social support; ERL, exercise rehabilitation literacy.

### Sufficiency analysis

Based on the results of the necessity analysis, this study developed truth tables for each of the two aforementioned groups to examine the condition grouping patterns associated with various anticipated outcomes. In accordance with criteria proposed by Fiss (2011), the consistency threshold in the truth table was established at a minimum of 0.80, and the proportional reduction in inconsistency (PRI) threshold was set at no less than 0.75. Considering both the number of available cases and the interpretability of the results, the frequency threshold was uniformly set at 1 for both the low-adherence and high-adherence groups in this study.

The sufficiency analysis generates three types of solutions-complex, intermediate, and parsimonious-based on different treatments of logical remainders. The complex solution includes all observed combinations of antecedent conditions without any simplification, resulting in a large number of configurations that are often difficult to interpret. The parsimonious solution incorporates both empirical evidence and logical remainders to identify the most essential antecedent conditions, referred to as core conditions, which cannot be excluded under any simplification. The intermediate solution refines the parsimonious solution by integrating theoretical and substantive knowledge to guide the inclusion or exclusion of logical remainders through counterfactual reasoning. Conditions that appear only in the intermediate solution are identified as peripheral conditions. Among the three, the intermediate solution is generally preferred for its balance between empirical grounding and theoretical plausibility, offering the greatest explanatory value. Consequently, this study presents the configuration of exercise adherence antecedent conditions in CHF patients, utilizing the classical framework of core, peripheral, and irrelevant conditions. As illustrated in Table 6, the analysis initially identified a total of seven distinct solutions. According to the criteria proposed by Ragin (1987), configurations should meet a consistency threshold greater than 0.75 and a coverage threshold above 0.25. After applying these criteria, L3 was excluded due to insufficient coverage. Consequently, four configurations associated with high exercise adherence and two configurations associated with low exercise adherence were retained in the final analysis. The overall consistency and coverage for the configurations leading to high exercise adherence are 0.774 and 0.854, respectively, indicating that these four configurations account for 85.4% of the cases. Conversely, the overall consistency and coverage for the configurations associated with low exercise adherence are 0.929 and 0.399, respectively, suggesting that these two combinations of influencing factors account for 39.9% of the cases.

**Table 6:** Configurations of high/low exercise adherence in CHF patients.

COM-B	Conditions	High-adherence				Low-adherence	
		H1	H2a	H2b	H3	L1	L2
Motivation	Perception of benefits	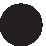	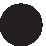	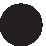	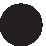	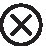	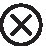
	Kinesiophobia	–	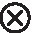	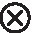	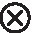	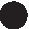	–
	Exercise self-efficacy	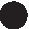	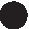	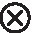	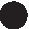	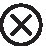	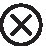
Capability	Exercise rehabilitation literacy	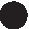	–	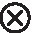	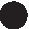	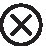	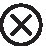
Opportunity	Exercise prescription	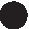	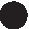	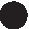	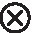	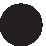	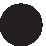
	Multiple social support	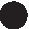	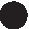	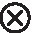	–	–	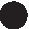
	Raw coverage	0.542	0.520	0.479	0.327	0.308	0.318
	Unique coverage	0.079	0.032	0.119	0.101	0.020	0.015
	Consistency	0.866	0.831	0.806	0.798	0.939	0.968
	Overall coverage	0.854				0.399	
	Overall consistency	0.774				0.929	

“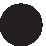,” presence as a core condition; “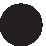,” absent as a core condition; “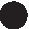,” presence as a peripheral condition; “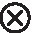,” absent as a peripheral condition; The short horizontal lines represent ambiguous condition.

Within the configurations associated with high exercise adherence, perception of exercise benefits consistently emerges as a core condition. This solution is further subdivided into four distinct configurations-H1, H2a, H2b, and H3-based on variations in the presence or absence of peripheral conditions.

In configuration H1, perception of exercise benefits emerged as the core condition, while exercise self-efficacy, exercise rehabilitation literacy, exercise prescription, and multiple social support functioned as peripheral conditions. Notably, all five conditions were present in the solution, with none being absent, suggesting a highly saturated and synergistic pathway toward high exercise adherence.

Configurations H2a and H2b represent two distinct variants of the solution H2, both leading to high exercise adherence but through differing patterns of condition combinations. In H2a, a comprehensive motivational profile is present, including a strong perception of exercise benefits, high exercise self-efficacy, and the absence of kinesiophobia. These motivational factors are supported by external opportunity conditions, namely a standardized exercise prescription and multiple social support. This configuration reflects a highly supportive and synergistic pathway, in which both internal and external facilitators are fully mobilized.

By contrast, H2b represents a more minimalist but sufficient configuration. In this pathway, perception of exercise benefits remains the core condition, while exercise self-efficacy and social support are absent, and kinesiophobia is also absent as a peripheral condition. The only opportunity-related factor present is the exercise prescription, serving as basic structural support. This indicates that for a subset of patients, a clear recognition of the value of exercise, unimpeded by psychological resistance, may be sufficient to maintain adherence, even in the absence of broader motivational or social resources. The contrast between H2a and H2b highlights the configurational diversity through which high adherence can be achieved either via a resource-rich pathway or a motivation-driven, structurally minimal alternative.

Configuration H3 is centered on the perception of exercise benefits, which appears as a core condition, while exercise self-efficacy and exercise rehabilitation literacy are present as peripheral conditions. In contrast, kinesiophobia and the exercise prescription are absent as peripheral conditions. This configuration suggests that when internal motivation is strong and supported by sufficient cognitive and informational resources, patients may still achieve high exercise adherence despite the absence of exercise prescription and psychological resistance. H3 thus represents a cognitively supported, motivation-driven pathway, in which behavioral adherence is enabled by individual knowledge and confidence, rather than by structural or external support.

Within the configurations associated with low-adherence, exercise prescription presence as a core condition, while the core conditions absent were perception of benefit, exercise self-efficacy, and exercise rehabilitation literacy. This solution is further subdivided into two configurations-L1 and L2-based on the status of the peripheral condition.

Configuration L1 is characterized by the presence of exercise prescription as a core condition, while three key motivational and capability-related factors-perception of exercise benefits, exercise self-efficacy, and exercise rehabilitation literacy-are absent as core conditions. Additionally, kinesiophobia is present as a peripheral condition, and social support is absent. This configuration represents a pathway where, despite having formal structural support, the lack of internal motivation and capability may prevent patients from translating guidance into action. The presence of kinesiophobia further reinforces psychological resistance, contributing to low adherence.

Configuration L2, on the other hand, also includes exercise prescription as a core condition, and shares the absence of perception of benefits, exercise self-efficacy, and rehabilitation literacy as core deficiencies. However, in this configuration, kinesiophobia is absent, and social support is present as a peripheral condition. This suggests a different dynamic in which some external support is available and psychological barriers are reduced, yet the absence of internal readiness and belief systems still limits adherence. Together, L1 and L2 highlight that external structural support alone is insufficient to promote exercise behavior when essential motivational and capability components are missing. Detailed metrics of consistency, overall coverage, and the raw and unique coverage for configurations are presented in Table 6.

## Discussion

This study employed a mixed-method approach integrating LPA and fsQCA to explore the mechanism of exercise adherence among CHF patients. LPA identified three distinct adherence profiles highlighting substantial interindividual variability. Building on this classification, fsQCA revealed four configurations associated with high adherence and two configurations associated with low adherence. Notably, the perception of exercise benefits consistently emerged as a core condition across all high-adherence configurations, while exercise prescription was present in both low-adherence configurations despite poor behavioral outcomes. These findings underscore the configurational and asymmetric nature of exercise adherence determinants and demonstrate that similar outcomes can arise from multiple distinct pathways.

The identification of perception of exercise benefits as a necessary condition across all high-adherence configurations underscores its fundamental role in initiating and sustaining exercise behavior among patients with CHF (Malone et al., 2012). Cardinal et al. have similarly noted that a pivotal moment in the behavioral change process occurs when individuals perceive that the benefits of exercise outweigh its barriers (Cardinal J et al., 2004). Within the framework of fsQCA, a necessary condition does not guarantee an outcome, but its absence renders the outcome nearly impossible. This is consistent with our finding that none of the patients exhibiting high adherence lacked benefit perception. While the perception of benefits alone may not be sufficient to achieve sustained adherence, it serves as a prerequisite motivational foundation, without which other facilitating conditions may not be effectively activated. This perspective aligns with most behavior change models, in which behavioral intention is considered a precursor to action. However, benefit perception is more closely aligned with the broader construct of motivation, and growing evidence points to a discrepancy between intention and actual behavior (Feil et al., 2023). Moreover, the predictive power of intention in explaining behavioral execution remains inconclusive. Translating motivation, shaped by benefit perception, into consistent behavioral engagement remains challenging—particularly in CHF populations where physical limitations, fatigue, dyspnea, and fear of movement often result in short-term rather than sustained participation in exercise rehabilitation (Nelson et al., 2023).

The four configurations associated with high exercise adherence exhibit strong theoretical alignment with the COM-B model, which posits that behavior arises from the dynamic interplay among capability, opportunity, and motivation. Although the COM-B model has been extensively applied to identify and categorize determinants of health behaviors (Yang et al., 2024; Young-Silva et al., 2024), few studies have explicitly examined how these three components interact configurationally to produce behavioral outcomes. Our findings illustrate that while comprehensive integration of capability, opportunity, and motivation clearly facilitates optimal adherence, certain partial combinations of these elements can also be sufficient to support adherence behavior.

Configuration H1 is primarily driven by reflective motivation, with opportunity and capability conditions working synergistically to support behavior. It showed the highest raw coverage among all high-adherence configurations, indicating that many patients achieve adherence through this comprehensive behavioral structure. This reflects an optimal scenario for behavior change, where full alignment of the COM-B components facilitates high adherence. Consistent with Paterson et al., who found that targeting at least two COM-B components is effective for improving physical activity (Paterson et al., 2024), this highlights the importance of comprehensive interventions. However, such ideal conditions are often difficult to achieve in practice, especially in resource-limited settings (Ferrel-Yui et al., 2024). While H1 offers a valuable benchmark for intervention design, flexible and adaptive strategies remain essential to address varying clinical realities. This is also supported by the relatively low unique coverage of Configuration H1, suggesting that its distinctiveness is limited and that it is not irreplaceable.

Both H2a and H2b represent motivation-dominant, opportunity-assisted configurations, where motivation serves as the core driver of exercise adherence, and external opportunities act as supportive rather than essential conditions. The key distinction lies in the level of opportunity provided: H2a reflects adherence behavior in a relatively well-supported context, whereas H2b represents a minimalist structure relying solely on a single opportunity element—namely, the exercise prescription. This contrast highlights a gradient in patients’ dependence on environmental resources, indicating that similar adherence outcomes can be achieved under varying degrees of structural support. A comparative analysis of the coverage shows that H2a and H2b exhibit similar raw coverage, suggesting that both configurations contribute comparably to explaining high exercise adherence within the study population. However, the significantly higher unique coverage of H2b indicates that this configuration accounts for a more distinct subset of patients whose behavior cannot be explained by other pathways. In contrast, H2a shares a large proportion of its explanatory power with other configurations, limiting its distinctiveness. This pattern suggests that while both configurations are common among adherent individuals, H2b captures a more irreplaceable behavioral mechanism, particularly relevant for patients whose adherence is sustained by motivation and minimal structural support alone.

Configuration H3 represents a capability-compensated pathway, where behavior is supported by both reflective and autonomous motivation, along with internal resources such as exercise rehabilitation literacy, despite the absence of external opportunity conditions. Although its raw coverage is relatively low, its unique coverage is notable, suggesting that this configuration accounts for a distinct subgroup of patients who rely on internal cognitive and motivational resources to sustain adherence. Notably, this finding aligns with prior research indicating that once individuals establish a habitual exercise routine, the influence of external factors tends to diminish, and behavior becomes increasingly self-regulated (Malone et al., 2012).

To examine causal asymmetry, this study conducted a sufficiency analysis of antecedent condition combinations associated with low exercise adherence among CHF patients, yielding two distinct configurations: L1 and L2. The core absent conditions in both configurations were perceived exercise benefits, exercise self-efficacy, and exercise rehabilitation literacy, while exercise prescription emerged as a core present condition. The peripheral present conditions differed: L1 included fear of exercise, whereas L2 included multiple social support. These findings illustrate a key principle of causal asymmetry, which posits that the absence of an outcome is not simply the inverse of the conditions that lead to its presence (Cangialosi, 2023). This stands in contrast to traditional linear models, where predictors are assumed to exert symmetrical and continuous effects on outcomes. In our results, high adherence configurations required the presence of motivation-related conditions as a necessary or core condition. However, in low adherence configurations, the absence of those motivational and capability factors, even when structural support like exercise prescription was present, still led to poor adherence. This asymmetry highlights the non-linear and complex nature of health behavior: interventions that promote adherence cannot be designed merely by reversing the factors linked to non-adherence. Rather, it underscores the importance of tailored, configurational approaches that account for how different combinations of factors interact under varying contexts.

In conclusion, there is no singular model for promoting exercise adherence among patients with CHF; rather, the four pathways are emblematic of “divergent routes leading to the same endpoint.” Based on the dissolution of the high-adherence configuration and the confirmation of the low-adherence configuration, this study concludes that motivation are pivotal factor in both the initiation and maintenance of exercise. Furthermore, enhancing exercise adherence among the majority of patients with CHF necessitates multidimensional systemic interventions, which should be grounded in the establishment and reinforcement of motivation. Kaushal et al. identified that the factors of perceived benefit and self-efficacy are modifiable and responsive to intervention in ethnically diverse patients with CHF (Kaushal et al., 2023). This finding indicates that healthcare professionals’ interventions targeting patients’ reflexive motivation may be more effective than those focusing on autonomous motivation, such as habits, emotions, and impulses. Reflective motivation interventions are designed to enhance the patient’s conscious effort in making accurate assessments, formulating detailed plans, and making rational decisions regarding exercise rehabilitation. Simultaneously, owing to inter-individual variability, not all patients require the presence of all three factors to attain the desired behavior. Identifying patients who necessitate particular triggers to achieve adherence to exercise is a crucial component of the clinical intervention process. This approach facilitates the implementation of tailored interventions to optimize outcomes. Furthermore, the findings of this study demonstrate the existence of an asymmetric and complex causal relationship concerning exercise adherence behavior in patients with CHF. Specifically, the antecedent conditions and outcomes do not consistently manifest or vanish simultaneously, and various antecedent conditions exhibit differential effects when combined in distinct ways. This insight offers a novel perspective for future research on the mechanisms underlying the exercise adherence behavior.

### Limitations

This study investigates the mechanisms underlying exercise adherence in patients with CHF from a configuration perspective; however, similar to many empirical studies, it possesses certain limitations. First, this study employed a questionnaire-based methodology, relying on self-reported data from patients, which inherently introduces the potential for subjective bias. Second, this research employs a cross-sectional design, which limits its scope to examining the static relationship between antecedent conditions and exercise adherence. Future investigations should incorporate Time-Series qualitative comparative analysis (TS-QCA) to facilitate a more comprehensive exploration of the multifactorial and multi-trajectory mechanisms underlying exercise adherence, with particular attention to temporal effects (Hino, 2009). Third, the low-adherence configurations in this study accounted for only 39.9% of the cases, indicating that a substantial proportion of low-adherence behaviors remain unexplained in terms of their underlying mechanisms. This suggests the potential need for the inclusion of additional antecedent conditions in future research. Finally, the findings derived from qualitative comparative analyses possess limited generalizability, necessitating cautious interpretation of causal inferences in conjunction with quantitative research. Furthermore, future studies should consider expanding the sample size to enhance validation.

## Conclusion

This research examined the mechanisms underlying exercise adherence in patients with CHF grounded in the COM-B model through the application of qualitative comparative analysis. By adopting a configuration perspective that highlights the interactions among influencing factors, the study identified six pathways characterized by multiple equivalences, with comprehensive explanations provided for each pathway. The study indicates that healthcare providers should account for the diverse pathways of exercise adherence and the varying roles each factor plays among different patients when designing an intervention program. This approach aims to identify the critical factors and strategically allocate intervention resources to enhance patient adherence and optimize the outcomes of exercise rehabilitation.

## Data Availability

The raw data supporting the conclusions of this article will be made available by the authors, without undue reservation.
